# Association of Medication Use and 8-Year Mortality Risk in Patients With Parkinson Disease: Drug-Wide Trial Emulation

**DOI:** 10.1212/WNL.0000000000213783

**Published:** 2025-07-11

**Authors:** Julia A. Tuominen, Trond Riise, Julia Romanowska, Mario H. Flores-Torres, Marianna Cortese, Clemens R. Scherzer, Kjetil Bjornevik, Jannicke Igland

**Affiliations:** 1 Department of Global Public Health and Primary Care, University of Bergen, Norway;; 2 Department of Epidemiology, Harvard T.H. Chan School of Public Health, Boston, MA;; 3 Department of Nutrition, Harvard T.H. Chan School of Public Health, Boston, MA;; 4 Stephen and Denise Adams Center for Parkinson's Disease Research, Yale School of Medicine, New Haven, CT;; 5 Department of Neuroscience, Yale School of Medicine, New Haven, CT;; 6 Department of Neurology, Yale School of Medicine, New Haven, CT; and; 7 Department of Genetics, Yale School of Medicine, New Haven, CT.

## Abstract

**Background and Objectives:**

There are currently no treatments that can halt or slow the progression of Parkinson disease (PD). The aim of this study was to identify new drug repurposing candidates for PD among existing prescription drugs that could be used to modify the disease course.

**Methods:**

This nationwide observational cohort study (2004–2020) used Norwegian health registries and was conducted as a high-throughput drug screen using an emulated target trial design. All individuals who met our prescription-based classification criteria for PD, were older than 25 years at the time of diagnosis, and were not prescribed the target drug in the past 2 years were included. We emulated a target trial for any drug filled by a minimum of 100 individuals at any pharmacy in Norway, which amounted to a total of 219 drugs. Mortality was used as an outcome to indicate disease progression. We estimated the effect of drug initiation, an observational analog of the intention-to-treat effect, on the 8-year risk of death, comparing initiators of the target drug with initiators of drugs within the same Anatomical Therapeutic Chemical classification system level 1 group. Inverse probability of treatment weighting was used to adjust for potential confounders.

**Results:**

The study included 14,289 individuals with PD (mean age 72 at diagnosis, 59% male) and identified 23 drugs associated with reduced mortality risk at 8 years. These drugs included ranitidine (histamine-2 blocker); pantoprazole and esomeprazole (proton pump inhibitors); losartan (angiotensin receptor blocker); atorvastatin (for high cholesterol); tadalafil (for erectile dysfunction); levothyroxine sodium (thyroid hormone); phenoxymethylpenicillin, erythromycin, and azithromycin (antibiotics); 4 nonsteroidal anti-inflammatory drugs; combined codeine/paracetamol and tramadol (opioid analgesics); zopiclone and melatonin (sleep aids); mianserin (antidepressant); mometasone (nasal corticosteroid); 2 opium-derived cough medicines; and dexamethasone (ophthalmologic corticosteroid).

**Discussion:**

Our study identified several drugs with potential disease-modifying properties that could be candidates for future clinical trials. It highlights the potential of repurposing existing medications to advance drug development. While these findings are exploratory and, therefore, insufficient to justify immediate clinical application, they warrant further investigation and potential inclusion in clinical trials.

## Introduction

While current treatments for Parkinson disease (PD) alleviate symptoms, no drugs exist to halt or slow its progression. Drug repurposing involves using approved drugs for new indications, offering a cost-effective and efficient alternative to traditional drug development.^1^ Its potential impact is underscored by the fact that, in 2024, 38% of new therapeutics for PD in clinical trials were repurposed.^2^ Conducting clinical trials of candidate drugs is, however, challenging because these trials require large sample sizes, long follow-up, and a large amount of resources.

Observational studies using health databases are valuable for uncovering potential repurposing opportunities because they allow for extensive follow-up of large samples to investigate medication history and disease outcomes.^3^ However, compared with randomized controlled trials (RCTs), observational studies are more prone to bias and sometimes address research questions that may not easily apply to clinical settings, which may affect the validity and interpretation of results.^4^ Emulating a target trial by applying RCT design principles to the analysis of observational data forces investigators to state trial criteria and critical assumptions.^5^ This has been shown to mitigate common biases encountered in observational studies and improves the translation of the results to real-world trials.^6^ A recent large-scale emulation study demonstrated a strong concordance between results from database studies and corresponding RCTs.^7^

To identify drugs with the potential for repurposing as new treatments for PD, we conducted a nationwide study on prescription drug use and 8-year mortality risk. We applied the emulated target trial framework and conducted a high-throughput, hypothesis-free screen of all drugs on the Norwegian market initiated by individuals after the diagnosis of PD.

## Methods

### Data Sources

This register-based observational cohort study included the entire Norwegian population in 2004, comprising almost 4.5 million individuals. Data from 3 Norwegian nationwide registries were used: the Norwegian Prescription Database (NorPD) (2004–2019),^8^ the Norwegian Patient Registry (NPR) (2008–2020),^9^ and Statistics Norway (SSB) (2004–2020). Registration in these registries is mandatory, without the option of withdrawing personal information, which ensures full coverage of all individuals in the specified period. The unique, pseudonymized identifiers enabled cross-linking of data between the registries. NorPD contains information on all prescriptions filled at any pharmacy in Norway, including corresponding Anatomical Therapeutic Chemical (ATC) codes, dispensing dates, and reimbursement codes.^8^ The ATC coding system, managed by the World Health Organization, classifies drugs hierarchically based on the anatomical and therapeutic properties of their active substances (eTable 1). Prescriptions that are partly or fully covered by the national insurance system, including those for chronic diseases such as PD, have an associated reimbursement code for the indication of use. NPR contains information from specialist health care providers on all inpatient and outpatient visits, including diagnoses coded according to the International Classification of Diseases (ICD), and medical procedures.^9^ Evaluation and diagnosis of PD are conducted within specialist health care. Specialists in the private sector who have reimbursement contracts with public health care are also mandated to report to NPR.^9^ Information on educational level, birth year, sex, emigration year, and death month and year up to 2020 was obtained from SSB.

Health care in Norway is government-funded and covers all lawful residents in the country from birth. Children receive health care for free while adults pay a small fee up to a certain annual limit.^10^ Hospitalizations are free of charge for all residents.

### Inclusion Criteria

To be included in a drug trial, individuals were required to be diagnosed with PD for less than 5 years, to be at least 25 years of age at diagnosis, and to have not been prescribed the target drug for 2 years preceding drug initiation (eTable 2). One individual could participate in multiple trials of different drugs.

Case identification was based on data from NorPD because of the longer follow-up period compared with NPR while diagnostic information from NPR was used to validate the prescription-based classification criteria. Individuals met the criteria for PD if they had either (1) ≥ 4 prescriptions of monoamine oxidase B (MAO-B) inhibitors (ATC group N04BD) or (2) ≥ 4 prescriptions of levodopa along with at least 1 PD reimbursement code (ICD-10: G20). We required the PD reimbursement code to exclude other conditions treated with levodopa because 28% of individuals prescribed levodopa did not have a G20 diagnosis in NPR. Reimbursement codes and data from NPR indicated that MAO-B inhibitors were very specific for PD treatment, and in Norway, the drug is indicated for PD specifically. Only 23% of individuals who had been prescribed dopamine agonists had a G20 diagnostic code in NPR, indicating low positive predictive value, which is why this drug group was not incorporated in the classification criteria. Of those meeting our prescription-based PD criteria between 2009 and 2019, 92% were recorded in NPR at least once with G20 as the primary diagnosis, underscoring the high specificity of the criteria. The positive predictive value of our classification criteria was 0.86 and the sensitivity was 0.87 when compared with a requirement of ≥2 G20 codes in NPR.

We ensured the inclusion of only incident PD cases by excluding patients with any antiparkinsonian prescription in 2004. The date of PD diagnosis was set to the date of the last prescription required for meeting our PD criteria.

### Intervention and Outcomes

For each trial, the initiation of the target drug was considered as the baseline of treatment intervention. Individuals with a record of at least 1 filled prescription of the target drug in NorPD between the date of diagnosis and 2019 were assigned to the treatment group of the intervention. Drugs were analyzed individually at ATC level 5. To mitigate confounding by indication,^11^ we mimicked an active-comparator design by assigning to the control group individuals prescribed any drug within the same anatomical main group (ATC level 1) as the target drug. We randomly selected 10,000 control initiations if the number of all control initiations exceeded that number. Examples of a target drug and associated control drugs are provided in eTable 3. Individuals receiving the target drug, and therefore, already in the target arm, were excluded from the control arm. Initiations of drugs within the same pharmacologic subgroup (ATC level 3) were also excluded. Antiparkinsonian drugs (ATC prefix N04) were not included in the analyses. The outcome of interest was the 8-year risk of death after drug initiation, indicated by the date of death recorded in SSB up to 2020.

### Covariates

The following covariates were considered as potential confounders because of their possible association with both drug initiation and mortality: sex, educational level at the time of drug initiation, age at the date of PD diagnosis, age at the time of drug initiation, calendar year of drug initiation, level of comorbidity, and the 20 most common comorbidities in our PD population (eTables 4 and 5). We used the RxRisk comorbidity index accommodated to the Norwegian population and treatment practices to adjust for comorbidity levels.^12^ It categorizes prescription drugs into 46 chronic health conditions based on the ATC classification system, which can be used in the absence of complete diagnostic data. Individual-specific comorbidity indices were calculated by summing the weights of baseline comorbidities, determined by the reported strength of association of each comorbidity category with one-year mortality.^12^ In addition to the RxRisk index, we included the 20 most common comorbidities as binary variables. An individual was assigned a value of 1 for each comorbidity present during the two-year period before initiation and 0 if the comorbidity was not present.

### Statistical Methods

**Figure 1:**
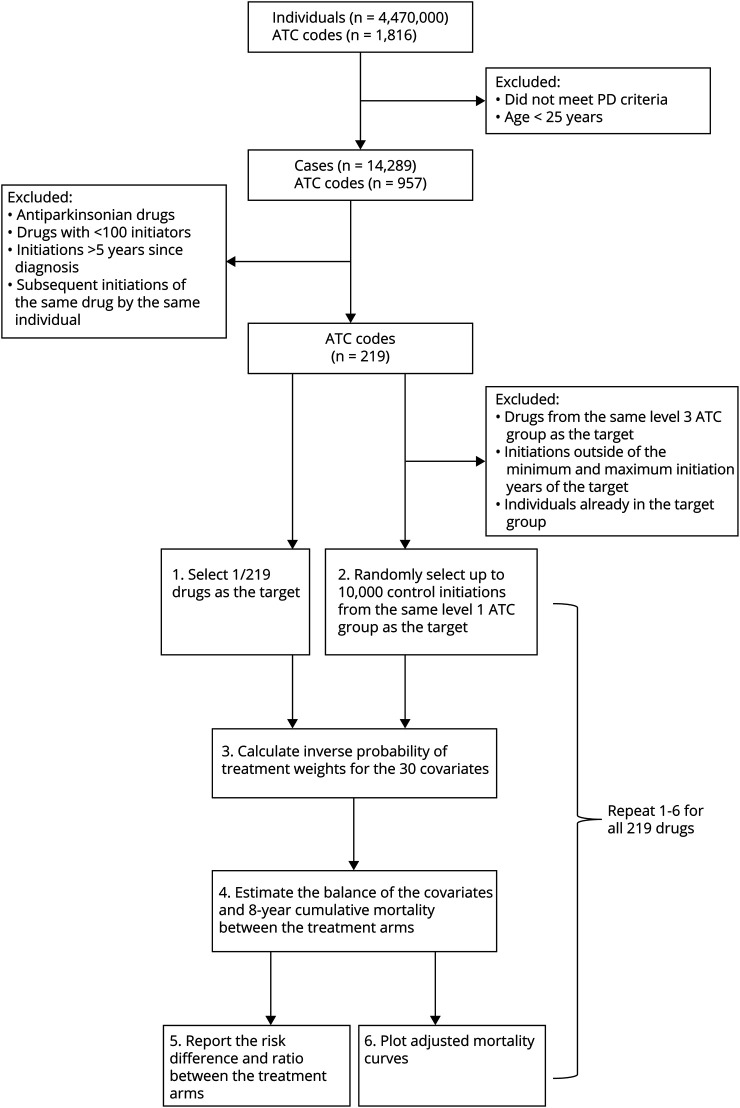
Flowchart of Exclusion Criteria and Analysis Steps

We emulated a target trial for each drug prescribed to at least 100 individuals with PD during the study period, following the protocol specified in eTable 2. The effect of interest was the effect of drug initiation, a close observational analog of the intention-to-treat effect. We only included control initiations within the same range of calendar years as the target drug prescriptions. Our analytical strategy is illustrated in Figure 1. The time line of data collection and follow-up is displayed in eFigure 1.

We assumed treatment groups to be exchangeable at baseline, conditional on the covariates. We used stabilized inverse probability of treatment weighting (IPTW) to adjust for confounding by these covariates.^13^ Missing information on educational level was categorized separately while the other variables had no missing data. Age at diagnosis and age at drug initiation were included as continuous variables in the logistic regression model used to estimate the weights. A standardized mean difference of less than 0.1 was required for a variable to be considered balanced between the treatment arms, and we calculated the percentage of covariates meeting this criterion.

Follow-up started from the date of drug initiation and ended at death or censoring (at 8 years after the trial start, date of migration, or administrative censoring in February 2020), whichever occurred first. We estimated the 8-year risk of death for each treatment group using a pooled logistic regression model.^14,15^ Time was modeled in months with linear and quadratic terms, including interaction terms between intervention and time, weighted for the 30 covariates. Bootstrapping with 500 samples was used to derive 95% CI and *p* values for the risk difference and risk ratio between the intervention arms. Confounder-adjusted survival curves were estimated using the IPTW Kaplan-Meier method.^16^

We ran several sensitivity analyses. To determine the extent to which unsatisfactory covariate balance influenced the results, we included any unbalanced covariates in the regression model in addition to the IPTW. In the second sensitivity analysis, we aimed for stricter control of confounding by indication by selecting controls from the same ATC level 2 group (example in eTable 3). We conducted 3 sensitivity analyses to test confounding by contraindication, which may arise if people considered too fragile are not prescribed certain drugs. First, we restricted the sample to individuals younger than 75 years at drug initiation, and in a separate analysis, we restricted the sample to individuals with a comorbidity index below 7 (the median across all initiations). To test whether some drugs are associated with lower mortality as a function of not being prescribed to individuals close to death, we applied a time lag of 1 year between drug initiation and the date of death. To test the robustness of the results when using a different age limit, we restricted the sample to individuals aged 65 or older at diagnosis.

Finally, to explore whether drugs associated with reduced mortality in the PD population were also associated with mortality risk in the general population, we tested these drugs in a random sample of 10% of the Norwegian population aged 65 or older on January 1, 2006. Initiations of control drugs were randomly selected with a 1:4 ratio to the target drug initiations. We followed the same analysis pipeline as in the main analysis. The covariates included in the IPTW were sex, education, age at drug initiation, year of drug initiation, RxRisk index, and the 20 most common comorbidities in this population.

We report drugs that were associated with reduced risk of death at 8 years in the main analysis, with a nominal *p* value less than 0.05 and at least 90% of the covariates balanced. We also report *p* values corrected for multiple hypothesis testing using the Benjamini-Yekutieli procedure, which accounts for correlations among the prescription drugs.^17^

All the analyses were performed in R version 4.3.0. IPTWs were created with the WeightIt package, version 0.14.2.^18^ Adjusted survival curves were created with the adjustedCurves package 0.10.1^19^ and plotted with ggplot2 3.4.2.^20^ Bootstrapping was performed with the boot package 1.3–28.1.^21^

### Standard Protocol Approvals, Registrations, and Patient Consents

The project received ethical approval and exemption from informed consent from the Western Norway Regional Committee for Medical and Health Research Ethics (REK West: 2017/1508). Permissions to use and merge data were obtained from NorPD, NPR, and SSB.

### Data Availability

The individual-level data used in this study are protected by the Norwegian Personal Data Act and can only be accessed by applying to the data holders through helsedata.no after approval by the Norwegian Regional Ethics Committee. Full results from all analyses are available at github.com/jtu009/PD_progression_screen.

## Results

Of the 14,289 individuals older than 25 years and diagnosed with PD during the study period, 59% were male. The mean age at diagnosis was 72 years (SD: 10). The maximum follow-up time was 16 years. During the study period, 41% of the individuals died, with a median survival time after diagnosis of 8 years. After diagnosis, at least one non-antiparkinsonian drug was initiated by 14,059 individuals. The screening included a total of 219 unique drugs and 131,760 drug initiations. An example of baseline descriptive characteristics of a trial (before IPTW) is provided in eTable 5. Estimated treatment duration since initiation is illustrated in eFigure 2.

Potential confounders were balanced in 193 of the 219 trials in the main analysis, in which 23 drugs were associated with decreased mortality at 8 years with a nominal *p* value less than 0.05 (Table). Covariate balance for the 23 drugs before and after IPTW is given in eTable 6. These included a histamine-2 blocker (ranitidine) and 2 proton pump inhibitors (pantoprazole and esomeprazole) for reflux disease; angiotensin receptor blocker (ARB) losartan for hypertension; atorvastatin for high cholesterol; phosphodiesterase-5 inhibitor tadalafil for erectile dysfunction; the thyroid hormone levothyroxine sodium; the antibiotics phenoxymethylpenicillin, azithromycin, and erythromycin; 4 nonsteroidal anti-inflammatory drugs (NSAIDs) (piroxicam, meloxicam, naproxen, and glucosamine); 2 opioid analgesics (combined codeine/paracetamol and tramadol); the sleep aids zopiclone and melatonin; the antidepressant mianserin; the nasal corticosteroid mometasone; ethylmorphine with and without a combined expectorant for cough; and the ophthalmologic corticosteroid dexamethasone. The unadjusted estimates are provided in eTable 7.

**Table:** Drugs Associated With Reduced Mortality Risk in People With PD

ATC code	Active ingredient	No. of persons		8-Year mortality risk (95% CI)		Risk difference (95% CI)	*P* value^a^	NNT	Risk ratio (95% CI)
		Target	Control	Target	Control	Events/100 persons			
				Events/100 persons					
A02BA02	Ranitidine	360	10,000	62.7 (54.9–69.5)	70.5 (68.9–71.7)	7.7 (0.8–15.2)	0.026	13	0.89 (0.79–0.99)
A02BC02	Pantoprazole	1,673	8,538	65.4 (61.7–68.8)	69.7 (68.0–71.3)	4.3 (0.6–8.3)	0.018	23	0.94 (0.88–0.99)
A02BC05	Esomeprazole	764	9,130	65.2 (60.3–70.0)	70.8 (69.1–72.1)	5.6 (0.6–10.9)	0.018	18	0.92 (0.85–0.99)
C09CA01	Losartan	281	10,000	51.6 (43.6–60.1)	63.5 (62.0–64.6)	12.0 (2.9–19.9)	0.010	8	0.81 (0.69–0.95)
C10AA05	Atorvastatin	811	8,268	54.5 (48.6–60.6)	64.9 (63.0–65.9)	10.4 (4.0–15.9)	<0.001*	10	0.84 (0.75–0.94)
G04BE08	Tadalafil	545	2,288	39.8 (31.9–47.2)	49.1 (45.5–52.6)	9.3 (1.3–17.3)	0.038	11	0.81 (0.65–0.97)
H03AA01	Levothyroxine sodium	175	1738	46.1 (37.1–54.3)	64.3 (61.3–67.7)	18.2 (9.8–27.2)	<0.001*	6	0.72 (0.58–0.85)
J01CE02	Phenoxymethylpenicillin	3,119	8,385	57.2 (55.2–59.2)	62.3 (60.5–63.4)	5.1 (2.5–7.1)	<0.001*	20	0.92 (0.89–0.96)
J01FA01	Erythromycin	660	10,000	57.8 (52.7–62.4)	63.9 (63.4–66.0)	6.1 (2.6–12.2)	0.010	17	0.91 (0.81–0.96)
J01FA10	Azithromycin	273	10,000	52.0 (42.9–61.6)	64.1 (63.2–65.7)	12.1 (2.8–21.8)	0.008	8	0.81 (0.66–0.96)
M01AC01	Piroxicam	235	2072	41.3 (29.8–54.2)	59.0 (55.3–62.0)	17.7 (3.8–28.1)	0.008	6	0.70 (0.52–0.93)
M01AC06	Meloxicam	136	2082	46.2 (35.2–59.7)	60.7 (57.3–63.9)	14.5 (0.7–26.2)	0.046	7	0.76 (0.58–0.99)
M01AE02	Naproxen	587	1992	48.5 (41.8–55.1)	59.0 (55.2–62.5)	10.6 (3.3–17.2)	<0.001*	9	0.82 (0.71–0.94)
M01AX05	Glucosamine	204	2069	44.7 (34.9–54.3)	60.0 (56.3–63.3)	15.3 (5.7–24.6)	0.010	7	0.75 (0.59–0.90)
N02AJ06	Codeine and paracetamol	3,227	10,000	58.7 (56.1–60.9)	66.6 (64.4–67.4)	8.0 (4.5–10.2)	<0.001*	13	0.88 (0.85–0.93)
N02AX02	Tramadol	2,407	10,000	60.6 (57.5–63.4)	65.4 (63.3–66.2)	4.8 (1.3–7.6)	0.008	21	0.93 (0.88–0.98)
N06AX03	Mianserin	663	10,000	57.9 (52.8–63.2)	64.4 (63.4–66.3)	6.5 (1.9–11.8)	0.008	15	0.90 (0.82–0.97)
R01AD09	Mometasone	584	8,110	49.5 (43.6–55.6)	56.4 (54.5–57.8)	6.9 (0.5–12.3)	0.030	14	0.88 (0.78–0.99)
R05DA01	Ethylmorphine	1706	6,340	49.9 (46.7–53.2)	58.5 (55.8–59.7)	8.7 (4.5–11.2)	<0.001*	12	0.85 (0.81–0.92)
R05FA02	Ethylmorphine and expectorant	404	9,020	49.5 (42.1–56.2)	56.5 (54.6–57.9)	7.0 (0.4–14.1)	0.038	14	0.88 (0.75–0.99)
S01BA01	Dexamethasone	531	7,321	52.9 (45.1–61.7)	62.4 (60.7–64.0)	9.6 (0.5–17.2)	0.032	10	0.85 (0.72–0.99)

Abbreviations: ATC = Anatomical Therapeutic Chemical; NNT = number needed to treat; PD = Parkinson disease.

Note: Risk differences and ratios were estimated with pooled logistic regression adjusted for sex, educational level, age at the date of PD diagnosis, age at drug initiation, year of drug initiation, comorbidity index, and the 20 most common comorbidities present among the PD population.

a *p* Values are for the risk difference estimate. *p* Values that were significant after correction for multiple testing are marked with an asterisk.

When we included the unbalanced covariates in the regression model in addition to IPTW, the risk difference was increased for tadalafil and attenuated for atorvastatin, azithromycin, and glucosamine. The risk difference for the other drugs remained materially unchanged (eTable 8).

When controls were selected from the same second-level ATC group, the risk difference was increased for tadalafil, the antibacterials, zopiclone, melatonin, mianserin, and the cough medicines (eTable 9). The risk differences were decreased for losartan, the combination of codeine and paracetamol, and tramadol. Mometasone was no longer associated with death in this analysis. The sample sizes were insufficient for the reflux disease drugs, atorvastatin, levothyroxine sodium, and the 4 NSAIDs.

In the analysis restricted to individuals younger than 75 years, all 23 drugs remained associated with reduced mortality (eTable 10). The risk differences increased for pantoprazole, the macrolides, piroxicam, naproxen, and melatonin but decreased for atorvastatin, tadalafil, glucosamine, and zopiclone. Less pronounced decreases or increases were observed for the remaining drugs.

In the analysis restricted to individuals with a comorbidity index below the median, the risk difference was substantially increased for ranitidine and azithromycin (eTable 11). Mometasone was no longer associated with mortality while the other drugs remained associated with reduced mortality risk. However, there was a marked covariate imbalance for tadalafil, levothyroxine sodium, and glucosamine in this analysis, preventing a proper comparison between the intervention arms.

When we applied a time lag of 1 year, there were only small changes in the effect estimates (eTable 12).

When limiting the lower age at diagnosis to 65, all drugs remained associated with reduced mortality, but we observed some changes to the effect estimates (eTable 13).

The general population aged 65 years or older included 790,743 individuals, with a median age of 75 years. Three of the 23 drugs identified in the initial analyses on PD (tadalafil, mianserin, and dexamethasone) were not associated with mortality in this population while the magnitude of association was weaker for 10 of the drugs (losartan, levothyroxine sodium, the antibiotics, piroxicam, meloxicam, naproxen, and the opioid analgesics) compared with the PD population (eTable 14). We observed larger effect sizes in the general population compared with the PD population for the reflux disease drugs, melatonin, mometasone, and the cough medicine ethylmorphine.

**Figure 2:**
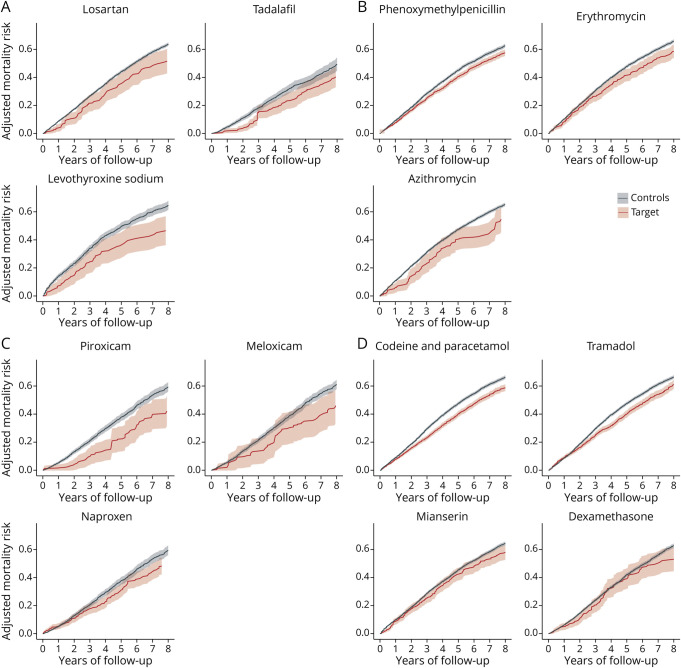
Eight-Year Adjusted Mortality Curves for Drugs Associated With Reduced Mortality Risk in the PD Population, With Weaker or No Association in the General Population (A) ATC level 1 groups C (cardiovascular system), G (genitourinary system), and H (hormonal preparations). (B) ATC level 1 group J (anti-infectives for systemic use). (C) ATC level 1 group M (musculoskeletal system). (D) ATC level 1 groups N (nervous system), R (respiratory system), and S (sensory organs). ATC = Anatomical Therapeutic Chemical; PD = Parkinson disease.

The adjusted mortality curves for the drugs with a more pronounced association with mortality in the PD population compared with the general population are displayed in Figure 2. The adjusted mortality curves for the drugs with similar or larger effect sizes in the general population are displayed in eFigure 3.

## Discussion

We conducted a high-throughput screening of medications initiated after PD diagnosis and estimated the 8-year risk of death for 219 drugs using an emulated target trial framework. We identified 23 drugs that were statistically significantly associated with a lower absolute risk of death. All drugs but 1 (mometasone) remained associated with reduced mortality in sensitivity analyses. Many of the identified drugs regulate and modulate biological pathways relevant to PD.

A central pathologic feature of PD is mitochondrial dysfunction.^22^ Seven of the identified drugs may directly influence this mechanism, including 2 macrolide antibiotics, 4 opioid derivatives, and melatonin. The macrolide antibiotics erythromycin and azithromycin were associated with a lower mortality risk in this study. Azithromycin was recently identified in another high-throughput target trial emulation as reducing falls and psychosis, but not dementia, as proxies for PD progression.^23^ Macrolides may decrease markers of aging and enhance the elimination of aging, pro-inflammatory cells by inhibiting mitochondrial metabolism.^24,25^ Even short-term exposures to antibiotics may cause long-term changes in the gut microbiome,^26^ which has been implicated in the pathogenesis and potential treatment of PD.^27^ If such long-term consequences also extend to other physiologic effects of these drugs, it enhances the biological plausibility of a disease-modifying effect despite brief treatment durations. Moreover, macrolide derivatives that retain the immunomodulatory and neuroprotective properties without antibacterial effect could be considered for repurposing while avoiding antibiotic resistance concerns.^28^ Four opioid derivatives, including 2 analgesics and 2 cough medicines, were also associated with reduced mortality. The dysfunction of the innate opioid system in PD has long been appreciated.^29^ The inhibitory influence of the activation of opioid receptors on the basal ganglia circuitry has been shown to be neuroprotective, likely through restoring mitochondrial membrane potential that reduces mitochondrial injury and by promoting clearance of damaged mitochondria.^30^ It is important to note that macrolides and cough medicines were also associated with reduced susceptibility for PD in our previous study using NorPD data.^31^ Furthermore, the circadian rhythm hormone and sleep aid melatonin was also associated with reduced mortality risk. Melatonin functions as a potent antioxidant by scavenging free radicals, stimulating antioxidant enzymes, and supporting efficient energy metabolism in mitochondria, where it is produced in high amounts.^32^

We identified several other drugs that have previously been suggested as disease-modifying in PD, or that are otherwise linked to PD. ARBs, of which the drug losartan was associated with a lower mortality risk in our study, were associated with reduced PD risk in 2 recent cohort studies^33,34^ and in our previous screening study.^31^ Consistently, the ARB valsartan was associated with reduced falls and psychosis in PD in a previous trial emulation.^23^ Losartan attenuated PD-like symptoms, oxidative stress, and dopaminergic neuron loss in rodents.^35^ We also identified 4 NSAIDs, the association of which with PD has been inconsistent in previous studies.^36,37^ Although a trend toward reduced PD risk has been reported for naproxen in NorPD data, no clear associations were found for any specific NSAID type.^38^ One study of modest sample size found no association between NSAIDs and PD progression.^39^ We also identified levothyroxine sodium for hypothyroidism, a condition that may be linked to higher PD risk.^40,41^ Mianserin, the only antidepressant associated with reduced mortality in our study, distinguishes itself from other antidepressants by effectively and selectively elevating norepinephrine levels by both blocking its reuptake and stimulating its release.^42^ The degeneration of the locus coeruleus and the following low levels of norepinephrine produced by that brain structure precede the degeneration of the substantia nigra, the hallmark of PD pathology.^43^ Mianserin was not associated with mortality in the general population, which could be consistent with a PD-specific effect.

The phosphodiesterase-5 inhibitor tadalafil for erectile dysfunction, which was associated with reduced mortality risk in people with PD in our analyses, has neuroprotective effects on dopaminergic neurons and seems to reverse cognitive dysfunction in rodents.^44,45^ There is also emerging evidence of improved cognition in individuals with mild cognitive impairment and reduced risk of Alzheimer disease in response to phosphodiesterase-5 inhibitors.^46,47^ Alternatively, the use of tadalafil in the PD population may reflect better overall health and survival, rather than a genuine causal effect. By contrast, in the general population, where tadalafil use was associated with higher mortality risk, use of tadalafil may reflect poorer health. The reason for the seemingly reduced mortality in response to an ophthalmologic corticosteroid in the PD population, but not in the general population, can only be speculated on. While locally acting drugs seem unlikely to be neuroprotective, when the blood-brain barrier is compromised, as in PD,^48^ eye drops may have systemic side effects through several routes of absorption.^49^

Our study has several strengths. We used nationwide registries that enabled a large sample size and comprehensive drug exposure assessment with minimal missing information. The relatively long follow-up time allowed us to examine mortality as an outcome, which is likely to reflect disease progression rather than mere symptom amelioration. Furthermore, the study included the entire Norwegian PD population, the results, therefore, being reasonably generalizable to populations with similar demographic structure and health care systems, such as other Nordic countries.

Our study also has some limitations. We did not have access to clinical progression scores or causes of death and, therefore, relied on all-cause mortality risk as an indicator of disease progression. Consequently, some of the results may not reflect disease modification but rather effects on general mortality, which we explored in the general population. Because aging is the strongest risk factor of PD, some pathophysiologic processes may be common to both PD and aging. This overlap may explain the observed associations in both populations, although most showed stronger effect sizes in the PD population. It is possible that drugs that displayed equally strong or a stronger association with mortality in the general population compared with the PD population do not have a PD-specific effect. However, results from our emulated trials in the general population must be interpreted with caution because factors affecting both drug initiation and mortality are arguably more diverse and complex than among patients with PD and would require a finer level of assessment not available in registry data. Furthermore, we lacked information on clinical diagnostic markers that could be used for case validation. Despite the high congruence between our prescription-based algorithm and G20 diagnostic codes, the inclusion of some non-PD cases is still possible. Owing to lack of information on factors that predict treatment discontinuation and on medication use during hospitalizations and stays in nursing homes, we could not estimate per-protocol effects. We also lacked information on over-the-counter drugs (including naproxen ≤250 mg and glucosamine of the identified drugs), which could result in effect attenuation. Furthermore, although the emulated target trial approach minimizes common biases in observational studies, the possibility of confounding by unknown and unmeasured variables is still present. For instance, the diagnosis itself and the severity of the disease affect the behavior of the patient and the treating physician. This creates a complex relationship between the disease process and medication patterns that may be challenging to disentangle in observational studies. We conducted 3 sensitivity analyses to reduce the potential influence of confounding by contraindication, yet the results remained largely unchanged. Many of the identified drugs may be used in the treatment of nonmotor symptoms, such as gastrointestinal dysfunction, sexual dysfunction, pain, sleep disturbances, and depression.^50^ The quality of health care as an unmeasured variable could create an indirect relationship between the extent to which such symptoms are attended to and overall mortality. Furthermore, the symptoms that dominate the clinical profile may vary across PD subtypes with different rates of disease progression, potentially creating indirect associations.

In this nationwide screening study emulating RCT design principles, we identified several drugs that were associated with reduced risk of death among individuals with PD. Among the identified drugs were macrolides, opioids, and melatonin, all of which have been demonstrated to influence mitochondrial quality-control mechanisms, which are central to the suggested pathologic origins of PD, but also to aging in general. Several of the identified drugs have previously been suggested to modify the course of PD, such as losartan and NSAIDs, or are linked to neuroprotective and immunomodulatory effects in general. We also identified multiple novel candidates with potential for drug repurposing as disease-modifying therapeutic in PD, including tadalafil, mianserin, macrolides, and opioid derivatives. Although these preliminary findings are insufficient to justify immediate clinical use, they warrant further investigation and potential consideration for future clinical trials.

## Glossary

ARB: angiotensin receptor blocker
ATC: Anatomical Therapeutic Chemical
ICD: International Classification of Diseases
IPTW: inverse probability of treatment weighting
MAO-B: monoamine oxidase B
NorPD: Norwegian Prescription Database
NPR: Norwegian Patient Registry
NSAID: nonsteroidal anti-inflammatory drug
PD: Parkinson disease
RCT: randomized controlled trial
SSB: Statistics Norway
